# A measurement scale to assess responsive feeding among Cambodian young children

**DOI:** 10.1111/mcn.12956

**Published:** 2020-01-30

**Authors:** Ndèye S. Sall, France Bégin, Jérémie B. Dupuis, Jimmy Bourque, Lylia Menasria, Barbara Main, Lenin Vong, Vannary Hun, David Raminashvili, Chhorvann Chea, Lucie Chiasson, Sonia Blaney

**Affiliations:** ^1^ École des sciences des aliments, de nutrition et d'études familiales Université de Moncton Moncton New Brunswick Canada; ^2^ Programme Division (Early Childhood Nutrition) UNICEF New York, New York; ^3^ Faculté des sciences de l'éducation Université de Moncton Moncton New Brunswick Canada; ^4^ Royal College of Physicians and Surgeons of Canada Ottawa Ontario Canada; ^5^ Independent Consultant Mississauga Ontario Canada; ^6^ Independent Consultant Phnom Penh Cambodia; ^7^ World Vision Cambodia Phnom Penh Cambodia; ^8^ National Institute of Public Health (NIPH), Ministry of Health Phnom Penh Cambodia; ^9^ Ministère du développement social Miramichi New Brunswick Canada

**Keywords:** active feeding, Cambodia, measurement scale, responsive feeding, young children

## Abstract

The caregiver–child interaction during mealtime, which refers to responsive feeding (RF), influences child's dietary intake. In Cambodia, given the level of malnutrition, getting better knowledge of RF among young children is essential, but to do so, using an appropriate assessment tool is necessary. We aim to develop and to validate a measurement tool to assess RF in two different situations (before and after an intervention) among children 6–23 months old. This research is part of a larger trial assessing the impact of nutrition education combined or not with the provision of complementary foods on child nutritional status. The “Opportunistic Observation Form” from the Process for the Promotion of Child Feeding package was used to collect data on RF through direct observations of child's meal episodes. Data were used to define an initial scale composed of four constructs and 15 indicators. Confirmatory factor analyses (CFA) and Hancock and Mueller's *H* reliability indices were computed to assess the validity and reliability of the scale. The final tool was applied to baseline and endline data. At baseline, the sample included 243 pairs and, at endline, 248 pairs. The final scale included two latent constructs (RF and active feeding) that comprise three indicators for active feeding and five for RF. Criteria for fit indices of CFA were met for both constructs though better at baseline. Reliability coefficients were above 0.80 for each construct at baseline and endline. This research proposes a scale that could be used to assess active feeding and RF. Further validation is warranted in different contexts.

**Abbreviations**
UNICEF: United Nations Children's Fund
ProPAN: Process for the Promotion of Child Feeding
PAHO: Pan American Health Organization
WHO: World Health Organization
NGO: non‐governmental organization
NE: nutrition education
SPSS: Statistical Package for the Social Sciences
CFI: comparative fit index
TLI: Tucker–Lewis index
RMSEA: root mean square error of approximation
MMI: model modification index
*df*: degree of freedom
SRMR: standardized root mean square residual

**Key messages**

- For optimal nutritional status and development, responsive feeding practices are essential among young children.
- More research is needed to develop valid and reliable measurement tools to assess responsive feeding.
- This paper use data from two cross‐sectional surveys to develop and to validate a measurement tool and then, to assess responsive feeding among Cambodian children 6‐23 months.
- Our study proposes a scale that could be used to assess active feeding and responsive feeding but further validation is warranted in different contexts.

## 1 INTRODUCTION

Child malnutrition is of concern in low‐ and middle‐income countries, and it is well recognized that one of its major causes relates to inappropriate feeding practices (Development Initiatives, 2017; United Nations Children's Fund [UNICEF], 1990; World Health Organization [WHO] & UNICEF, 2003). Yet, although complementary foods should be of optimal quality, density, and of age‐appropriate frequency on a daily basis, caregiver–child interactions during mealtime also exert a significant influence on the child's dietary intake, growth, and development (Engle & Pelto, 2011; Moore, Akhter, & Aboud, 2006; Pan American Health Organization [PAHO] & WHO, 2003; Pelto, Levitt, & Thairu, 2003; Spill et al., 2019; Vazir et al., 2013; WHO, 2005).

In the 1990s, WHO and UNICEF have recognized that the child nutritional status was not only related to food availability but also to access to care (WHO & UNICEF, 2003). The UNICEF conceptual framework on determinants of child nutritional status considers feeding practices as one of the core components of care affecting nutritional status through their influence on dietary intake and health status (UNICEF, 1990). WHO and UNICEF defined 12 care practices essential for child survival. One of them, “active feeding,” was defined as an alternative to a passive, nonresponsive feeding style that was associated to low intake and inadequate growth (WHO & UNICEF, 2003). The concept of active feeding was subsequently extended to responsive feeding (Birch, 1998; Engle & Pelto, 2011; WHO & UNICEF, 2003).

Different definitions of responsive feeding have been proposed to help at evaluating its various components. Moore et al. (2006) refer to responsive feeding as a behaviour that is sensitive and responsive to the child's signals and psychomotor abilities at meal time, providing a stimulating but supervised, structured and nondistracting environment during meals (Moore et al., 2006). The authors also make a distinction between responsive and active feeding. The latter is defined as a behaviour that encourages the child to eat or the mother to feed. More recently, Pérez‐Escamilla, Segura‐Pérez, and Hall Moran (2019) defined responsive feeding as “feeding practices that encourage the child to eat autonomously and, in response to physiological needs, which may encourage self‐regulation in eating and support cognitive, emotional, and social development” thereby including the concept of active feeding. In the United States, the Healthy Eating research project of the Robert Wood Johnson foundation defines responsive feeding as a process that involves reciprocity between the child and caregiver during the feeding episode (Robert Wood Johnson Foundation, 2017).

Indicators and criteria have been developed to evaluate various components of responsive feeding, but constructs and indicators developed by Moore et al. (2006) have been used in several settings (Abebe, Haki, & Baye, 2017; Aboud, Moore, & Akhter, 2008; Aboud, Shafique, & Akhter, 2009; Mwase, Mutoro, Owino, Garcia, & Wright, 2016). They encompass the following components: responsive and active feeding, self‐feeding, and the distracting feeding situation. WHO and PAHO have also provided guidelines on responsive feeding (PAHO & WHO, 2003; WHO, 2005). Yet, in spite of the above efforts, as pointed out recently by Pérez‐Escamilla et al. (2019), still, limited attention has been dedicated to “how to feed children.”

As part of their initiative to promote optimal child feeding (Process for the Promotion of Child Feeding [ProPAN]), PAHO, WHO, and UNICEF (2013) have developed a form to collect data on responsive feeding. To our knowledge, the ProPAN observation form has not been validated yet nor has a scheme been developed to organize data collected with this tool.

Responsive feeding has been assessed in low‐ and middle‐income countries. Research has focused on gestures and vocalizations of the mother or the child during meals, including encouragements (Abebe et al., 2017; Alvarez, Wurgaft, & Wilder, 1982; Bentley, Stallings, Fukumoto, & Elder, 1991; Dearden et al., 2009; Gittelsohn et al., 1998; Ha et al., 2002; Moore et al., 2006; Mwase et al., 2016; Wondafrash, Amsalu, & Woldie, 2012), food acceptance (Ha et al., 2002; Moore et al., 2006; Aboud et al., 2008 & 2009; Dearden et al., 2009; Wondafrash et al., 2012; Mwase et al., 2016), help provided to the child (Abebe et al., 2017; Aboud et al., 2008; Dearden et al., 2009; Gittelsohn et al., 1998; Ha et al., 2002; Oni et al., 1991; Ruel, Levin, Armar‐Klemesu, Maxwell, & Morris, 1999; Wondafrash et al., 2012), self‐feeding (Moore et al., 2006, Aboud et al., 2008 & 2009; Dearden et al., 2009; Mwase et al., 2016; Abebe et al., 2017), or caregiver attitude (Dettwyler, 1986; Gittelsohn et al., 1998; Ha et al., 2002; Moore et al., 2006; Aboud et al., 2008 & 2009, Dearden et al., 2009; Wondafrash et al., 2012; Abebe et al., 2017) and the management of food refusal (Ruel et al., 1999; Ha et al., 2002; Moore et al., 2006; Aboud et al., 2008 & 2009, Wondafrash et al., 2012; Abebe et al., 2017). Moreover, several studies were conducted in an intervention context, which makes it difficult to isolate the impact of responsive feeding on child dietary intake (Gittelsohn et al., 1998; Ha et al., 2002; Aboud et al., 2008 & 2009; Dearden et al., 2009; Bentley, Wasser, & Creed‐Kanashiro, 2011). To advance responsive feeding research and practice, more clarity is needed in measurements (Black & Hurley, 2017). A recent systematic review of instruments assessing these practices among children 0–5 years of age highlights the limited testing of validity and reliability of instruments that have been used as well as the fact that few were tested among children under 2 years of age (Heller & Mobley, 2019).

In Cambodia, as well as in other low‐ and middle‐income countries, undernutrition is of concern. Stunting and wasting affect 32% and 10% of children below 5 years of age, respectively (National Institute of Statistics & ICF International, 2015). Appropriate complementary feeding practices remain limited as only 36% of children between 6 and 23 months of age benefit from the minimum acceptable diet. Food alone is not sufficient to ensure optimal nutrition (Black & Aboud, 2011), and responsive feeding is particularly relevant during complementary feeding as young children progress from a milk‐based diet to soft and solid foods and self‐feeding. However, despite the importance of responsive feeding for child's nutrition that could prevent both, under‐ and overnutrition, there is limited information on these practices in Cambodia. From what little information that can be gathered, feeding practices including responsive feeding appear to be nonoptimal (Food and Agriculture Organization, 2015).

Using data collected among a group of Cambodian children 6–23 months old with the ProPAN package opportunistic form, the aim of this study was to develop and to validate a measurement tool that could be used to assess responsive feeding in two different situations namely before and after an intervention. In other words, using indicators for which data were collected through the ProPAN form, our objective was to develop a procedure to analyse these data and, thereafter, to develop and to validate a scale that could be used to assess responsive feeding. To do so, data collected through a nutrition cross‐sectional survey at two time points were used. They originate from a larger study that intended to assess the impact of promoting appropriate feeding practices combined or not with the supply of local foods on child dietary intake and nutritional status.

## 2 METHODS

### 2.1 Study area

This study is part of a larger research that was conducted between February and August 2017 in the rural Soth Nikum district of Siem Reap province in Cambodia, where stunting and wasting affect 36% and 10% of children under 5 months old, respectively, and where only 36% of children aged 6–23 months old receive the minimum acceptable diet (National Institute of Statistics & ICF International, 2015). Mortality among children under 5 months old is 56/1,000 live births, which is above the national rate of 35/1,000 (National Institute of Statistics & ICF International, 2015).

### 2.2 Design and sampling

The research was a cluster‐randomized, controlled trial conducted in the intervention zone of an international non‐governmental organization (NGO) that aimed to assess the impact of nutrition education (NE) provided through counseling and group sessions combined or not with the provision of locally produced complementary foods (cricket and moringa powders) on child dietary intake and nutritional status and, more particularly, iron status. A total of 14 villages out of 298 were randomly selected in the NGO operational area. Each village was randomly assigned to one of the following groups: (a) provision of moringa powder combined with NE, (b) provision of cricket powder combined with NE, and (c) NE alone. The sample size was defined to detect a mean improvement of 1 g/dl of haemoglobin level among each group of young children between baseline and endline assuming 90% power and a 5% significance level. The sample included 360 children between the ages of 6 and 23 months spread throughout three experimental groups. For the current study, data were collected before the intervention (baseline) and the end of implementation (endline). More details on the research methodology can be found in a previous paper (Menasria et al., 2018).

### 2.3 Preparatory work

Before undertaking the data collection, 38 local enumerators were recruited and trained on the methodology and survey tools including the “Opportunistic Observation Form” from the ProPAN package (PAHO, WHO,, & UNICEF, 2013), which was intended to be used to collect data on responsive feeding.

During training, each item of the initial form from the ProPAN package was reviewed with all enumerators to ensure proper understanding. The following item was removed because its translation in Khmer proved to be problematic: “Is the child only served portions of the foods or drinks that are served to the rest of the family or are some foods or drinks prepared specially for the child?” The term “bottle” was removed from the item “Is a spoon, bottle, or other utensil used to feed the child” due to the pro‐breastfeeding Cambodian context. The item “Is the child breastfed to satiety?” was rephrased as follows: “Caregiver offers breast to the child” because the term satiety was unclear and subject to several interpretations by enumerators. Yet, despite the change in phrasing, offering the breast to the child was still considered a responsive feeding practice. When a mother breastfeeds responsively, she may offer her breast when her baby shows signs of hunger or when he is distressed (UNICEF, 2006).

The observation form was translated in Khmer and reviewed again to ensure its conformity with the English version. A pretest was conducted in villages located in the neighbouring communities of the training centre.

### 2.4 Data collection

At baseline and endline, data were collected using the final and pretested form, which initially included observations related to 15 indicators. Two direct observation periods were performed during a meal on different week days. There was a delay of 1 week between the two observation periods. In our context, we defined a meal as a dish of at least rice or porridge combined with any other foods (e.g., vegetable and fish).

The day before, the enumerator contacted the child's mother/caregiver to take an appointment and ask her/him at what time the mid‐day meal was supposed to be served to the child. Each enumerator was at home at a time suggested by the caregiver to enable the direct observation of a feeding episode. The recording of observations of the caregiver–child dyad feeding behaviours were done in a non‐intrusive manner.

Child dietary intake was assessed through three quantified 24‐hr recalls carried out during two different days of the week and one weekend day at baseline and endline but on different days than the data collection on responsive feeding behaviours. Quantities of foods daily consumed by children were estimated based on local utensils (bowls, cup, and spoons). Breastfeeding daily frequency was also recorded. Semi‐structured interviews were also conducted at baseline with the head of each household in presence of the child's caregiver, then privately with the child's caregiver. During the first interview, socio‐demographic data were collected on each household member (e.g., age, sex, and education), household access to a healthy environment and improved water source, housing conditions, assets ownership, and household food security. The second interview gathered data on health and feeding practices including child health status. The Demographic and Health Survey 2014 Cambodia questionnaire was adapted to collect the aforementioned data (National Institute of Statistics & ICF International, 2015).

### 2.5 Data analysis

#### 2.5.1 Definition of variables and constructs

In this study, we have extended the Moore et al.'s definition (2006) to also consider PAHO/WHO guidelines (2003, 2005) on responsive feeding namely breastfeeding on demand, having the child experimenting spoon and other utensils, and being responsive to child's hunger and need (e.g., serves additional portions to the child and foods/dishes only served to the child).

Active feeding refers to behaviours that encourage the child to eat either indirectly through verbal encouragements and games or directly through forcing the child and options in cases of child's refusals to eat (Moore et al., 2006; PAHO & WHO, 2003; PAHO, WHO, & UNICEF, 2013; WHO, 2005). Self‐feeding provides the child with opportunity to meet his hunger needs independently (Moore et al., 2006; PAHO & WHO, 2003; PAHO, WHO, & UNICEF, 2013; WHO, 2005). The distracting feeding situation refers to the caregiver's behaviour, which is directed toward a nonfeeding partner or event such as having a conversation with someone else (Moore et al., 2006).

Based on Moore et al.'s framework and the aforementioned definitions, data collected on each indicator were grouped under one of the following four constructs (Table 1): (a) responsive feeding (seven indicators), (b) active feeding (six), (c) self‐feeding (one), and (d) distracting feeding situation (one).

**Table 1:** Responsive feeding measurement scale: constructs, indicators and criteria

Construct	Indicator	Criteria*		
		Positive (+1)	Neutral (0)	Negative (‐1)
Responsive feeding	1. Caregiver offers the breast to the child	Yes		No
	2. Caregiver serves the child first	Yes	No	
	3. Child eats with caregiver and family members	Yes		No
	4. Food is served to the child on his own plate	Yes		No
	5. Spoon or other utensil is used to feed the child	Yes (6‐11 mos)	Yes (12‐23 mos)	No
	6. Food or drinks are only served to the child (not to other members of the family)	Yes (6‐11 mos)	Yes/No (12‐23 mos)	No (6‐11 mos)
	7. Caregiver serves additional portions to the child during the meal	Yes	No	
Active feeding	8. Caregiver is near the child and attentive during mealtime	Yes		No
	9. Caregiver talks to the child, verbally encourage him to eat	Yes		No
	If yes, what type of words are said:			
	‐ Positive wordings	Yes		No
	‐ Negative wordings	No		Yes
	10. Caregiver encourages the child when he is eating well	Yes		No
	11. Caregiver motivates the child to eat more using gestures/games or by demonstrating to him how to eat	Yes		No
	12. Caregiver physically forces the child to eat during the meal	No		Yes
	13. During the meal, the child refuses the food	No		Yes
	If yes, the caregiver:			
	‐ Encourages the child to eat	Yes		No
	‐ Threatens the child	No		Yes
	‐ Forces the child	No		Yes
	‐ Threatens and forces the child	No		Yes
Self‐feeding	14.‐Child feeds self without help from caregiver	Yes (12‐23 mos)		Yes (6‐11 mos)
	‐ Child mostly feeds self but receives help from caregiver	Yes (12‐23 mos)		Yes (6‐11 mos)
	‐ Child is mostly fed by the caregiver but sometimes feeds self	Yes (6‐8 mos)		Yes (9‐23 mos)
	‐ Child is fed only by caregiver (child does not touch food or utensils)	No (9‐23 mos)		Yes (9‐23 mos)
		Yes (6‐8 mos)		No (6‐8 mos)
Feeding situation	15. When the child is eating, the caregiver spends time:			
	‐ Eating own meal	No (6‐8 mos)	Yes (9‐23 mos)	
	‐ Taking care of other family members	No		Yes
	‐ Selling foods	No		Yes
	‐ Doing household tasks	No		Yes
	‐ Taking care of the child	Yes		No

#### 2.5.2 Data coding

Each observation was coded as positive, negative, or neutral even if observed once (Table 1). There was no count of observations. The codes were mutually exclusive. A score was given to each indicator and to each child as follows: −1 = *negative behavior*, 0 = *neutral behavior*, and +1 = *good/beneficial behavior.* A behaviour was considered positive if it promoted child food intake during the meal, whereas a behaviour was considered negative if it hindered food intake or even stopped it (Moore et al., 2006; PAHO, WHO, & UNICEF, 2013). A behaviour was considered neutral if it had no impact on food intake. For instance, if a child aged 12–23 months old did not have a utensil to eat with, given his ability to eat by himself at this age, it was assumed that not having a utensil would not increase or decrease his food intake and, thus, this behaviour was coded as neutral.

In the case of verbal encouragements, responses were grouped as positive or negative. Positive encouragements included words or statements from the caregiver such as “you are a good baby,” “you eat well,” and “it is good to eat well for your growth,” whereas wordings that were ordering suddenly, threatening, or scaring the child were considered discouragement (PAHO, WHO & UNICEF, 2013). If the child refused to eat, responses were grouped under the following: (a) positive if the caregiver encouraged the child to resume eating with positive words and (b) negative if the caregiver threatened and/or forced the child to eat (Table 1). With regards to utensils used to feed the child, selection of foods given to the child, self‐feeding, and feeding situation, each score was assigned based on the child's age (Table 1). All responses were again grouped as positive (+1), neutral (0), or negative (−1).

All data were entered in the SPSS software (SPSS Statistics for Windows, Version 26.0. Armonk, NY: IBM Corp.).

#### 2.5.3 Descriptive analysis

For baseline and endline data and for each indicator, frequency distributions were produced for all observed episodes. Indicators for which the frequency distribution of the baseline and endline data had more than 85% of responses/observations in one category were removed in subsequent analyses (Tabachnick & Fidell, 2013). These indicators were the following (Table 1): indicator #7: « Caregiver serves additional portions to the child during the meal » and indicator #8: « Caregiver is near to the child and attentive during mealtime ». However, the indicator related to the use of physical force by the caregiver to make the child eat during the meal was maintained given its potential impact on the child's dietary intake despite the fact that less than 10% of caregivers implemented this practice.

To further simplify the measurement tool, indicators of self‐feeding and distractive feeding situation were both moved under the responsive feeding construct as per Moore et al.'s (2006) proposed subcodification process, which classified both constructs either under responsive or active feeding in analyses.

For baseline and endline data, the mean of the two meal observation episodes was calculated for each child and each indicator.

#### 2.5.4 Confirmatory factor analyses

In this current study, confirmatory factorial analyses (CFA) were used to develop and to validate the measurement tool. This is a common procedure to assess the internal or latent structure of an instrument of a questionnaire (Conway & Huffcutt, 2003; Floyd & Widaman, 1995; Lewis, 2017). In our case, we used CFA to confirm the theory and the item structure for the latent constructs. Prior to the analyses, the database was checked for missing data. As the item scores were ordinal, all data were transferred from SPSS to the Mplus software (Version 8, Muthén & Muthén, Los Angeles, CA, 1998‐2017) to perform polychoric and tetrachoric correlations to investigate relationships between indicators and produce the correlation matrix that would be factorized. Polychoric correlations were conducted to assess relationships involving at least one polytomous ordinal variable. Tetrachoric correlations were performed to assess the association between two dichotomous variables. Thereafter, using baseline data, CFA were performed to confirm the model's structure. Four criteria were retained to assess the goodness of fit for each factorial model: (a) the chi‐squared test, where a non‐significant *p* value (>.05) indicates an appropriate model fit, (b) the comparative fit index (CFI) and the Tucker–Lewis index (TLI), which should both be above 0.90, and (c) the root mean square error of approximation (RMSEA), which should be below 0.08 (as it indicates an acceptable adjustement of the model), with a 95% confidence interval that is entirely below 0.10 (Caron, 2018). Several models were tested starting with model 0 which comprises all 13 indicators (#1–6 and 9–15). At each step, the chi‐squared goodness of fit and other fit indices were examined to assess model fit. The Wald test was also performed to assess if each indicator in a model added a statistically significant contribution to model fit; indicators with non‐significant *p* values (≥.05) were removed from the model in subsequent analyses (Gana & Broc, 2018).

To assess the stability of estimates across the time, the final model was also tested on endline data. All goodness of fit indices were again computed to assess the strength of the evidence for construct validity of test scores. To evaluate the construct reliability of the final models, Hancock and Mueller's *H* reliability coefficients were estimated using the items' standardized factor loading: If the value of the *H* was above 0.80, it was considered as satisfactory (Hancock & Mueller, 2001).

### 2.6 Ethical

The research protocol was approved by the National Ethics Committee for Health Research, Ministry of Health, Cambodia (367‐NECHR) and the Ethic Committee on Human's Research of the Université de Moncton, Moncton, New Brunswick, Canada (#1617‐037).

## 3 RESULTS

From the initial sample of 360 caregiver–child pairs, data for 75 and 79 pairs were not available at baseline and endline, respectively. For most of these pairs (45 and 49), no data were collected because the meal had already been provided to the child (80.2%) or the child was sleeping or playing (13.3%) during the enumerator's visit or for other reasons (6.5%). For the other 30 pairs, data were only available for one meal for the same reasons.

**Figure 1:**
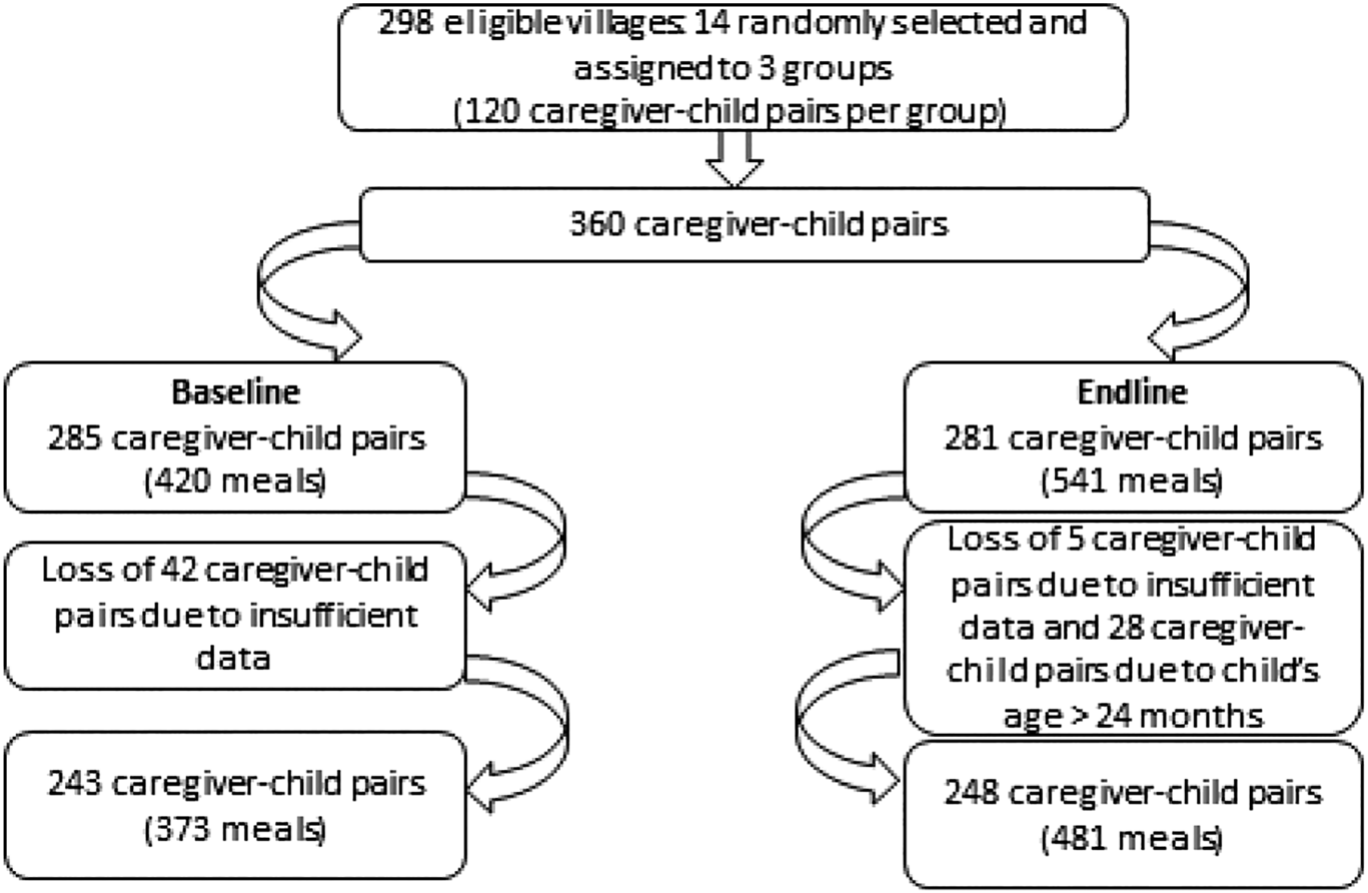
Flow chart describing study population at baseline and endline

At baseline, for the remaining 285 caregiver–child pairs, data were available for 420 meal episodes out of 570. Information for 150 meals was not available for the following reasons: The meal had already occurred (76.0%), the child was sleeping/playing during the enumerator's visit (5.3%), or other reasons (18.7%). In addition, at baseline, 48 meals were removed for a total of 43 caregiver–child pairs (38 pairs with 1 observed meal; 5 pairs with 2 observations) due to missing data (Figure 1). At endline, from the 281 caregiver–child pairs, data for five pairs were discarded for the same reason. In addition, given that the initial scale aims to measure responsive feeding among children 6–23 months, endline data on 28 child–caregiver pairs aged 24 months and above were also not considered for subsequent analyses (Figure 1). At baseline, the final sample included 243 pairs corresponding to 373 meal observations with 113 pairs having one observed feeding episode and 130 having two. At endline, 248 pairs were considered for a total of 481 meals, 39 pairs having one observed meal and 221 having two.

At baseline and endline, the majority of children were female (above 51%). Their mean age was 14.0 ± 5.0 months and 17.0 ± 4.8 months at baseline and endline, respectively (Table 2). Between baseline and endline, as shown in Table 3, there were significant difference between proportions for each indicator with the exception of indicators #13 (“options if the child refuses foods”) and #14 (“self‐feeding”).

**Table 2:** Frequency distributions (%) of data for each indicator of the responsive feeding initial scale at baseline (B) and endline (E)*

Construct	Indicator	# Obs	Criteria		
			Positive (+1)	Neutral (0)	Negative (‐1)
Responsive feeding	1.Caregiver offers the breast to the child	341/428 B/E	71.6/44.6*		28.4/55.4
	2.Caregiver serves the child first	332/436	43.1/73.6*	56.9/26.4	
	3.Child eats with caregiver and family members	335/481	45.7/39.9*		54.3/60.1
	4.Food is served to the child in his own plate	315/481	16.5/76.7*		83.5/23.3
	5.Spoon or other utensil is used to feed the child	307/480	17.6/10.4*	30.9/50.4	51.5/39.2
	6.Food or drinks are only served to the child (not to other members of the family)	314/481	8.0/1.9*	16.2/80.0	75.8/18.8
Active feeding	9.Caregiver talks to the child, verbally encourage him to eat	325/481	30.5/54.3*		69.5/45.7
	10.Caregiver encourages the child when he is eating well	329/481	30.7/52.8		69.3/47.2
	11.Caregiver ever motivates child to eat more using gestures/games or by demonstrating to him how to eat	329/481	9.1/32.4*		90.9/67.6
	12.Caregiver physically forces the child to eat during the meal	330/480	99.1/96.3*		0.9/3.8
	13. Options if the child refuses foods:	60/105			
	‐ Positive behavior		33.3/72.4		
	‐ Negative behavior				66.7/27.6
Self‐feeding	14. Having a:	279/479			
	‐ Positive behavior		41.9/31.5		
	‐ Negative behavior				58.1/68.5
Feeding situation	15.When the child is eating, the caregiver has an:	290/476			
	‐ Adequate behavior		47.9/79.2*		
	‐ Neutral behavior			34.5/0.0	
	‐ Inadequate behavior				17.6/20.8

* Indicate significant differences between proportions for each indicator (p<0.05).

**Table 3:** Description of the population at baseline and endline

Baseline/Endline			
Child	N	%	Mean (*SD*)
Sex	243/248	—	—
Male	111/121	45.7/48.8	—
Female	132/127	54.3/51.2	—
Age (months)	241/216	—	14.0 (5.0)/17.0 (4.8)
6–11	74/31	30.7/14.4	—
12–17	93/79	38.6/36.6	—
18–23	74/106	30.7/49	—

Results from the first CFA, which included all 13 indicators proposed in the initial scale, did not yield any values for the fit indices (Table 4). The Wald test values were non‐significant (*p* = .872) for indicator #12 (“caregiver forces the child …”) as well as for indicator #14 on self‐feeding (*p* = .820)

**Table 4:** Fit indices at baseline and endline

Index	Baseline						Endline		
	Model 0	Model 1	Model 2	Model 3	Model 4	Model 5	Model 4	Model 5	Model 5b
*X*^2^	128.51	111.32	94.20	68.39	38.15	29.07	88.35	87.52	46.96
*df*	64	43	34	26	19	18	19	18	18
*p*	.000	.000	.002	.000	0.006	.05	.00	.00	.00
RMSEA	0.07	0.08	0.09	0.09	0.07	0.05	0.11	0.12	0.07
CI 90%	0.05–0.08	0.07–0.10	0.07–011	0.06–0.11	0.04–0.10	0.01–0.09	0.09–0.14	0.09–0.14	0.05–0.10
*p*	.05	.003	.002	.011	.173	.426	.00	.00	.06
CFI	0.82	0.81	0.83	0.88	0.94	0.97	0.83	0.83	0.93
TLI	0.78	0.76	0.80	0.83	0.91	0.95	0.74	0.73	0.89
SRMR	0.09	0.10	0.08	0.08	0.07	0.05	0.07	0.07	0.05

Abbreviations: CFI, comparative fit index; CI, confidence interval; *df,* degree of freedom; RMSEA, root mean square error of approximation; SRMR, standardized root mean square residual; TLI, Tucket–Lewis index.

Model 1, which excluded indicators #12 and #14 was run. Results showed a significant *p* value for the chi‐squared test, whereas the RMSEA index was at 0.08 and the CFI and TLI were below 0.90 (Table 4). Model 2 was run after the removal of indicator #13 (“options if the child refuses foods”) given that the *p* value of the Wald test for this indicator was above 0.05 (*p* = 0.604). Again, as for Model 1, the results of the chi‐squared test, RMSEA, CFI, and TLI were unsatisfactory. Model 3 was run after the removal of indicator #6 because the *p* value of the Wald test was still above .05 (*p* = .255). The results show no improvement of the goodness‐of‐fit indices. Results of Model 3 were examined and indicator #1 (“caregiver offers the breast to the child”) was removed from the model. The value of the Wald test was at *p* = 0.169 for this indicator. Model 4 (which excluded indicator #1) was run and results show that values for all goodness‐of fit indices but the chi‐squared test were meeting recommended criteria. A final model (Model 5) was run in which a correlation between the errors of indicator #9 and #10 was added. This modification to the model was justified by the fact that the two indicators measure an analogous behaviour namely encouragements. All four criteria outlined to assess the goodness of fit of the model were met (Table 4).

In order to assess the stability of the final models, CFA of Models 4 and 5 were performed on endline data. The results did not show an acceptable model fit for any criteria (CFI, TLI, and RMSEA) for both models. Model 5b that incorporated a correlation between the errors of indicators #2 and #3 was then tested on the endline data set. Results showed a good fit for most indices with the exception of the chi‐squared test *p* value (*p* < .05) and the TLI that was at 0.89, marginally below the cut‐off of 0.90. (Table 4).

The final scale includes eight indicators divided in two constructs, namely responsive feeding (five indicators) and active feeding (three indicators). The range of the final score for each construct is from −4 to +4 (Table 5). Hancock and Mueller's *H* reliability coefficients for the final models were estimated on data for each construct at baseline and endline. For active feeding, *H* coefficients are 0.86 and 0.89 for baseline and endline, respectively. As for responsive feeding, *H* coefficients are 0.814 and 0.82 for baseline and endline, respectively.

**Table 5:** Responsive and active feeding measurement final scale

Construct	Indicator	Criteria		
		*Positive (+1)*	*Neutral (0)*	*Negative (‐1)*
Responsive feeding	2.Caregiver serves child first	Yes	No	
	3.Child eats with caregiver and family members	Yes		No
	4.Food is served to child on his own plate	Yes		No
	5. Spoon or other utensils is used to feed the child	Yes (6‐11 mos)	Yes (12‐23 mos)	No
	15. When child is eating, the caregiver spends time:			
	‐ Eating	No (6‐8 mos)	Yes (9‐23 mos)	
	‐ Taking care of other family members	No		Yes
	‐ Selling foods	No		Yes
	‐ Doing household tasks	No		Yes
	‐ Taking care of child	Yes		No
Active feeding	9.Caregiver talks to the child, verbally encourage him to eat	Yes		No
	If yes, caregiver uses:			
	‐ Positive wordings	Yes		No
	‐ Negative wordings	No		Yes
	10.Caregiver encourages the child when he is eating well	Yes		No
	11.Caregiver motivates the child to eat more using gestures/games or by demonstrating to him how to eat	Yes		No

## 4 DISCUSSION

Using data collected among a group of Cambodian children 6–23 months old through the Opportunistic Observation Form of the ProPAN package (PAHO, WHO & UNICEF, 2013), this research aimed to develop a scheme to organize the data and thereafter, to develop and to validate a simple measurement scale that could be used to assess responsive feeding in two different contexts namely before and after the implementation of an intervention. We used two different data sets (baseline and endline) from a larger research, which aimed to assess the impact of the promotion of optimal child feeding practices alone as well as combined with the provision of two local foods.

Based on Moore et al.'s framework as well as on PAHO/WHO's guidelines (Moore et al., 2006; PAHO & WHO, 2003; WHO, 2005), four constructs and related indicators were initially included in the scale. Data on each indicator part of the form were assigned to one of the four constructs and were coded based on available literature and subjective judgment. CFA were performed to confirm the theoretical model structure of the proposed scale or, in other words, to develop and to validate the scale.

In the first measurement model, two indicators were removed namely the use of force to feed the child and self‐feeding. Removing these indicators did not improve the goodness‐of‐fit of the model though. It is likely that the high frequency of positive observations (99%) and, thus, the heavily skewed distribution of data regarding the use of force make it difficult to obtain an acceptable confirmatory model (Floyd & Widaman, 1995). In fact, as a CFA is based on factorizing a variance–covariance (or correlation) matric, a low variability would translate into low variances and attenuate covariances/correlations, making the factor loading low and, thus, non‐significant. With regard to self‐feeding, it is possible that other indicators under the responsive feeding construct such as eating with the caregiver and family members, being served on his own plate and first, as well as being fed with a utensil, were more important in terms of variables measuring responsive feeding and supersede it.

In the subsequent model (Model 3), the indicator “foods, dishes, or drinks are only served to the child (not to others family members)” was removed. Again, as for the indicator on the use of force, the frequency distribution showed high proportions of observations under one criterion (Table 2). It is also possible that given the age of our population, which was above 12 months for two thirds of children, providing them with special foods was not so important because they were likely able to eat family foods.

In Model 4, the indicator on offering the breast was removed. As mentioned previously, the phrasing of the indicator on caregiver offering breast to the child was modified from the original form (“is the child breastfed to satiety”). The new formulation of this indicator may have affected its ability to reflect a responsive feeding behaviour.

The final scale included eight indicators and two constructs (responsive and active feeding) namely (a) whether the child was served first, (b) eats with his/her caregiver and other family members, (c) was served on his own plate, (d) was fed with a utensil, (e) was encouraged to eat, (f) was encouraged when he was eating well, (g) was motivated to eat by gestures and games, and (h) how the caregiver spent her time when the child was eating. The modelling process resulted in the refinement of the scale, which was also an objective of our process.

Even though, with baseline data, the final scale respects all four criteria to conclude about its validity (Caron, 2018; Gana & Broc, 2018), the application of the scale on endline data did not provide satisfactory results with regards to all defined four indices. In Australia, Jansen et al. have used CFA to develop a questionnaire for assessing feeding practices among children aged 2 years olds and as well as among thoses aged 2 to 5 years (Jansen, Mallan, Nicholson, & Daniels, 2014, Jansen, Williams, Mallan, & Nicholson, 2016). To conclude about its validity, similar indices to ours were used (chi‐squared test, the CFI and TLI, RMSEA, and weighted root mean square residual). Interestingly, the model fit was classified as “excellent” if it met the chi‐squared test criteria and all four others, “good” if the model did not fit the chi‐square tes but met the criteria of other indices, “adequate” if the model met the criteria for at least two indices, and lastly, “poor” if only one criterion was met. In our context, if we refer to Jansen et al.'s (2016) classification, the model fit was excellent with baseline data and adequate for endline.

Differences between the model fit at baseline and endline could be attributed to significant changes in terms of feeding practices. This may be partially attributed to the impact of the intervention (nutrition education through counseling and group session) on the caregiver's behaviour. Another explanation is that the adjustment of the baseline model was somewhat data‐driven, which would make the model vulnerable to overfitting (modelling the signal, but also a good part of the noise in that model). Although the “noise” is mostly random, its contribution to the model may not replicated in the endline model.

On the basis of the *H coefficient*, we can assert that the reliability of the measurement scale is good for both constructs given that all values were above 0.80. In other words, the measure has a set of items that represent the latent constructs.

Encouragements to eat seem to be important behaviours related to active feeding. In addition, having the mother or other family members near the child during the meal may increase the likelihood that the child will be encouraged or motivated to eat. If nearby the child, the caregiver or any other family member may be more attentive to the child's signals of hunger and satiety and, in return, may choose to encourage the child to eat by using verbal encouragement and games. However, this does not mean that the child will have a better nutritional status (Engle, Bentley, & Pelto, 2000).

Our results show that around 17% of children were eating on their own plates at baseline and 75% at endline probably because they were getting older at endline. Moreover, between 30% and 42% of caregivers had a positive behaviour regarding self‐feeding. Although different context, in Ethiopia, a study investigating feeding practices among a group of child aged 12–23 months found that only 45% of them were eating in their own plate and verbally encouraged to self‐feed (Spill et al., 2019). The discrepancy between our results and Ethiopia data may be due to the fact that our population included younger children.

As stated before, in our study, it is likely that changes between baseline and endline with regards to responsive and active feeding behaviours were somewhat attributed to the trial. Nutrition education that comprised a component on responsive feeding was provided through two face‐to‐face counselings and a group education session on a monthly basis. However, this hypothesis needs to be further investigated given that other factors besides NE such as the provision of food could have prompted responsive feeding behaviors. It is also possible that there was a better application of the form by enumerators from endline compared with baseline. Moreover, to assess dietary intake, 24‐hr recalls were performed at baseline and endline as well as on a weekly basis between both surveys. Therefore, it is possible that the increase in the aforementioned improvements regarding responsive and active feeding behaviors could be attributed to a higher attention of the caregiver to the child diet because they were asked to record food quantities at baseline and endline. Notably, recalls were done throughout the study, therefore potentially inducing the same bias. Lastly, with child aging, feeding practices have also likely evolved.

The CFA of our study suggest excellent fit indices for the proposed model at baseline. The final eight‐item structure of the scale may thus be used to assess caregiver–child eating behaviours in a non‐intervention area. However, caution is required regarding its use among a population who had benefited from an intervention on promotion of child feeding. The reliability of the scale to adequately measure each construct at both times is also satisfactory. Lastly, the proposed scale is a simple tool.

In spite of the aforementioned results, our study has some limitations. No causal inference can be performed due to the research design. Another limitation relates to the context. The scale has been developed from an Asian‐based sample as well as among a children population living in a low‐income country, which could limit its generalization to other cultural and socio‐economic settings. The methodology used to collect data may also have induced the Hawthorne effect, a bias in which individuals modify an aspect of their behaviour because they are observed (McCambridge, Witton, & Elbourne, 2014). A high proportion of observations of feeding episodes could not be made for various reasons. Yet it is difficult to assess whether or not this has induced bias. However, our sample size was sufficient for the purposes of the study at both periods of data collection. According to Floyd and Widaman (1995), having 5–10 participants per variable is adequate, whereas Gorsuch (1983) suggests a minimum of 200 as a total sample. Finally, given the objective of assessing responsive and active feeding practices is to ensure that they are optimal and that they contribute to the improvement of child dietary intake and nutritional status; investigating the relationship between these behaviours as measured with our proposed scale with young child dietary intake and nutritional status could further validate the operational constructs of the tool.

## 5 CONCLUSIONS

In conclusion, this research proposes a scale that could be used to assess active and responsive feeding in a non‐intervention and after‐intervention situations. However, it certainly deserves further validation in different contexts.

## CONFLICTS OF INTEREST

The authors declare that they have no conflicts of interest.

## CONTRIBUTIONS

NSS, SB, JBD, and JB analysed the data and did the interpretations. NSS wrote the first draft of the manuscript. LM, VH, DR, and LL were involved in the data collection. NSS, SB, BM, FB, and DR designed the research. All authors critically revised the manuscript. All authors also contributed to and approved the final version of the manuscript.
